# Suppression Method of Optical Noises in Resonator-Integrated Optic Gyroscopes

**DOI:** 10.3390/s22082889

**Published:** 2022-04-09

**Authors:** Xuebao Kuai, Lei Wei, Fuhua Yang, Wei Yan, Zhaofeng Li, Xiaodong Wang

**Affiliations:** 1 School of Microelectronics, University of Science and Technology of China, Hefei 230026, China; kuaixuebao@semi.ac.cn; 2 Center of Materials Science and Optoelectronics Engineering, University of Chinese Academy of Sciences, Beijing 100049, China; weilei@semi.ac.cn (L.W.); fhyang@semi.ac.cn (F.Y.); xdwang@semi.ac.cn (X.W.); 3 Engineering Research Center for Semiconductor Integrated Technology, Institute of Semiconductors, Chinese Academy of Sciences, Beijing 100083, China; yanwei@semi.ac.cn; 4 School of Microelectronics, University of Chinese Academy of Sciences, Beijing 100049, China; 5 Beijing Academy of Quantum Information Science, Beijing 100193, China; 6 Beijing Engineering Research Center of Semiconductor Micro-Nano Integrated Technology, Beijing 100083, China

**Keywords:** optical gyroscopes, optical noises, resonators, suppression technology

## Abstract

Resonator-integrated optical gyroscopes have advantages such as all-solid-state, on-chip integration, miniaturized structure, and high precision. However, many factors deteriorate the performance and push it far from the shot-noise limited theoretical sensitivity. This paper reviews the mechanisms of various noises and their corresponding suppression methods in resonator-integrated optical gyroscopes, including the backscattering, the back-reflection, the polarization error, the Kerr effect, and the laser frequency noise. Several main noise suppression methods are comprehensively expounded through inductive comparison and reasonable collation. The new noise suppression technology and digital signal processing system are also addressed.

## 1. Introduction

Optical gyros are all-solid-state devices and can handle an extensive rotational speed range [1,2]. The ring laser gyro (RLG) [2,3] and the interferometric fiber optic gyro (IFOG) [4] are the two most common optical gyros utilized in numerous applications. However, their volume and cost limit them from being used in applications that require tiny and light gyros. The resonator-integrated optic gyroscopes (RIOG) with a waveguide-type ring resonator (WRR) as a sensing component [5,6,7] are intended to offer a new device that takes advantage of integrated optics technology. The rotation readout is calculated from the resonant frequency difference between the clockwise (CW) and counter-clockwise (CCW) lightwaves propagating through the WRR [8,9]. RIOGs enable batch production by merging the WRR and other independent optical parts onto a shared substrate. Moreover, the low cost, small volume, high precision, and high degree of robustness can be realized.

The shot-noise of the photodetector determines the ultimate sensitivity of the RIOG. However, many characteristics, including backscattering [10,11,12,13,14,15,16,17,18,19,20,21,22], back-reflection [23,24,25,26,27,28], nonlinear Kerr effect [29,30,31,32,33,34,35,36,37], polarization fluctuation [38,39,40,41,42,43,44,45,46,47,48,49,50], and laser frequency noise [51,52,53], have negative impacts. These negative impacts degrade the performance of RIOGs and make it worse than the shot-noise limited theoretical sensitivity. IFOG using a broadband low-coherence source is an excellent way to solve many problems [54]. However, the RIOG necessarily uses a high coherent light source, and these various negative effects will be more serious. Other measures must be taken to suppress these negative impacts and make the signal processing in the RIOG more effective. Although the RIOG has great theoretical sensitivity, the existing performance of RIOG lags far behind IFOG. It is inspiring that numerous negative impacts restricting the sensitivity of the RIOG have been resolved one-by-one. The emergence and development of new technologies, including microsphere [55], the hollow-core photonic bandgap fiber (HC-PBF) [56,57,58,59], and the silicon-based photonic integration [60,61], have a significant impact on RIOGs. This paper reviews the approach to deal with the noises encountered in the RIOG, including the backscattering noise, the back-reflection noise, the polarization noise, the Kerr effect-induced noise, and the laser frequency noise. In Section 2, we show the basic signal detection system of the RIOG. Section 3 examines the causes of different noise generation and their corresponding noise suppression techniques in detail. Next, in Section 4, we show the new noise suppression technology and digital signal processing system in recent years. Then, we summarize the noise suppression technology in Section 5. Finally, in Section 6, the conclusions and the possible directions of future research are discussed.

## 2. The Basic Configuration of the RIOG

**Figure 1:**
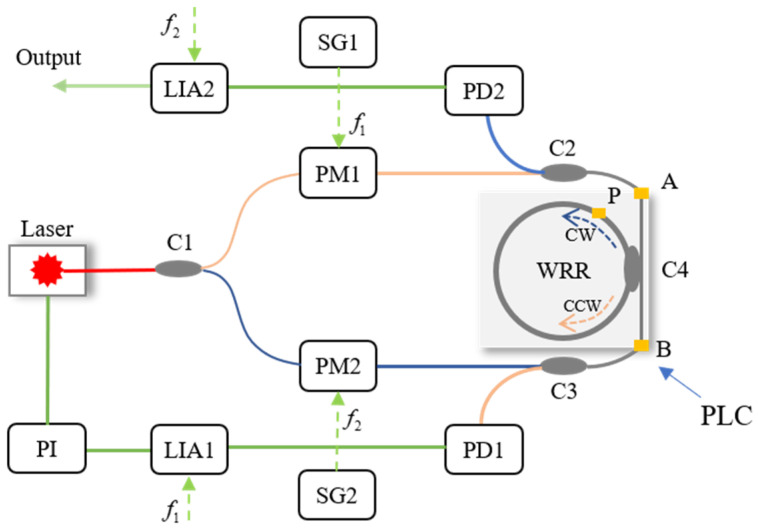
System structure of the RIOG based on single-phase modulation technology.

A basic signal detection system of the RIOG based on phase modulation technology is shown in Figure 1, including a laser, 3 dB couplers (C1, C2, C3), phase modulators (PM1, PM2), photodetectors (PD1, PD2), lock-in amplifiers (LIA1, LIA2), proportional-integral controllers (PI), and the WRR. Low-pressure chemical vapor deposition fabricates the WRR on a silicon planar light-wave circuit (PLC). The light source’s center wavelength is 1550 nm for the distributed feedback laser diode (DFB-LD). First, the PDs modulated the differential frequency of CW and CCW lights to eliminate backscattering noise. In order to reduce polarization noise, a polarization-maintaining silica PLC is used instead of the single-mode chip. The PLC includes two input and output directional couplers (C2 and C3) and a resonator coupler (C4). The coupling ratio of C1, C2, and C3 design couplers is 50%, and C4 is about 5%. It is optimized based on the total loss in the WRR, including propagation loss of WRR, excess loss caused by curvature, and excess loss through the coupler C4. Then, Sin waves with proper modulation frequencies (f1, f2) from signal generators SG1 and SG2 drive PM1 and PM2, respectively. The PD1 and PD2 detect the CW and CCW light from the resonator, respectively. The LIA1 receives the output of PD1 and feeds it back to the laser diode controller (LDC, inside the laser instrument) to lock the central frequency of DFB-LD at the resonant frequency of the CCW lightwave. The proportional-integral (PI) controller is inserted between LDC and LIA1 to reduce the error of the lock-in frequency. The readout of the rotation rate is the demodulated CW light-wave signal from LIA2.

## 3. Noise and Suppression

This section will introduce various noises and their corresponding suppression methods in RIOG, including the backscattering noise, the back-reflection noise, the polarization noise, the Kerr effect-induced noise, and the laser frequency noise.

### 3.1. Rayleigh Backscattering Noise

#### 3.1.1. The Intensity of Rayleigh Backscattering Noise

Rayleigh backscattering noise is mainly determined by materials’ processing defects and the lattice of waveguide materials [10]. The fundamental theory has been developed, and several works have been published to solve the Rayleigh backscattering noise in the resonant fiber optic gyroscope (RFOG) [10,62]. However, these theories have not been used for RIOG. RFOG using the super-luminescent diode can successfully decrease Rayleigh backscattering noise. However, the transmission loss of the WRR is thousands of times that of optical fiber, so the Rayleigh backscattering noise is nine orders of magnitude more than RFOG [12,63]. Therefore, backscattering noise has a more significant impact on RIOG performance [63]. Consequently, it is critical to quantitatively measure and reduce Rayleigh backscattering noise to obtain a high-performance RIOG.

Figure 1 depicts the basic structure of a RIOG. Taking Rayleigh backscatter noise into account, the detector intensities (PD1 and PD2) are represented as [10]:

(1) $$I_{D1}=I_{s,ccw}+I_{b,cw}+I_{i1}$$

(2) $$I_{D2}=I_{s,cw}+I_{b,ccw}+I_{i2}$$

where $I_{s,ccw}$ and $I_{s,cw}$ are the intensities of two counterpropagating source beams. The portions induced by Rayleigh backscatter are expressed as $I_{b,cw}$ and $I_{b,ccw}$, respectively. $I_{i1}$ stands for interference intensity between $I_{s,ccw}$ and $I_{b,cw}$, and $I_{i2}$ stands for interference intensity between $I_{s,cw}$ and $I_{b,ccw}$.

**Figure 2:**
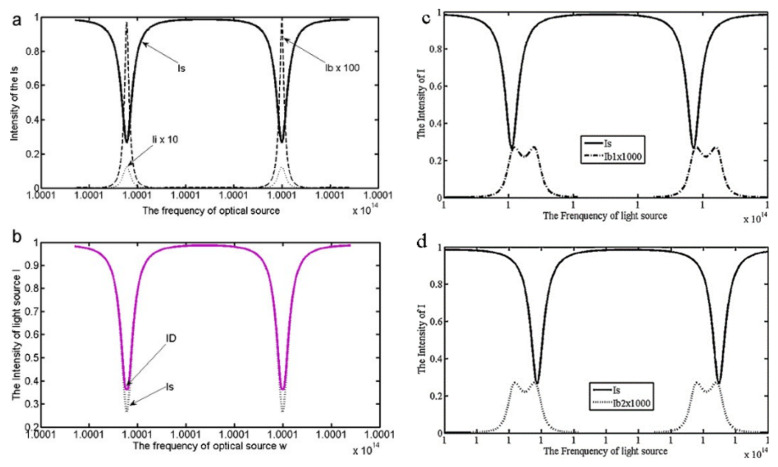
The intensity of Rayleigh backscattering noise. Where *I_s_* is the intensity of source beams, *I_b_* is the intensity of Rayleigh backscatter noise, *I_i_* represents interference intensity, and *I_D_* is the intensity of the detector. (**a**) The signal intensity of *I_s_*, *I_b_*, and *I_i_*. (**b**) The intensity of *I_s_* and *I_D_*. (**c**) Rayleigh backscattering noise of CW turn. (**d**) Rayleigh backscattering noise of CCW turn. Reprinted with permission from ref. [16]. Copyright 2011 Elsevier.

Feng et al. analyzed and evaluated the static and dynamic Rayleigh backscattering noise [16]. Under the static state, the intensity of Rayleigh backscattering noise and the interference intensity are shown in Figure 2a. Figure 2b shows the intensity obtained by the detector compared with the ideal intensity. The depth of resonance is decreased under the effect of Rayleigh backscattering. Meanwhile, the reduction of fineness influences the basic detection limit of RIOG. In the dynamic condition, as shown in Figure 2c,d, the intensity of Rayleigh backscattering noise will split into two peaks under the Sagnac effect. The symmetry of the resonance curve and the RIOG’s linear degree output will be reduced by the resonance characteristic.

#### 3.1.2. Suppression Method of Rayleigh Backscattering Noise in RIOG

The Rayleigh backscattering noise affects the basic detection limit and reduces the output linearity of the RIOG. Therefore, it is essential to suppress the Rayleigh backscattering noise in RIOG. There are three approaches to suppress the Rayleigh backscattering noise. The first approach uses binary phase-shift keying (BPSK) to reduce the impact of light interference. Takiguchi and Hotate et al. proposed that a BPSK scheme combined with an acousto-optic modulator (AOM) in RFOG could achieve carrier suppression of approximately 80 dB [11]. However, AOM is challenging to integrate for RIOG. The second is to delay the phase of one light beam before it transmits into the resonator. This method can be accomplished by inserting a Mach-Zehnder [64] switch before the resonator or allowing one light beam to transit a sufficiently long fiber. They are not conducive to integration for RIOG. The last is the phase modulation technique to modulate the two light beams with different frequencies in the resonator. The interference noise component in Equations (1) and (2) can be reduced by modulating and demodulating lightwaves at different frequencies.

Since Iwatsuki proposed the phase modulation technique to eliminate Ryleigh backscattering in 1984 [10], researchers have paid increasing attention to this technique. Single-phase modulation technology (SPMT) is the first generation of phase modulation technology, as shown in Figure 1. Several signal waves were selected to drive the PDs in RIOG, including sinusoidal wave [13,65], serrodyne wave [66,67,68,69], triangular wave [12,25], and trapezoidal wave [70].

**Figure 3:**
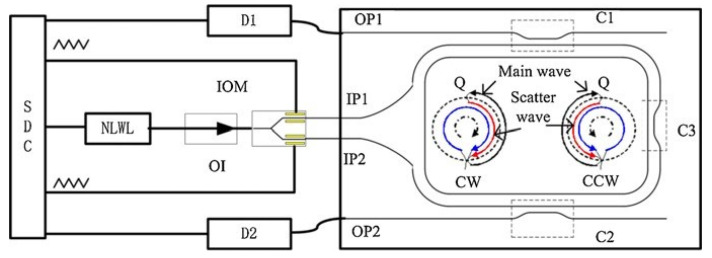
The configuration of the SPMT using the same triangular wave. SDC, NLWL, OI, IOM, OP1(2), IP1(2), and D1(2) represent the signal detecting circuit, laser, optical isolator, integrated optical modulator, output1(2), input1(2), and optoelectronics detector1(2), respectively. Reprinted with permission from ref. [16]. Copyright 2011 Elsevier.

Yu et al. presented a SPMT system with a 12.8 cm-long silica WRR using the triangular wave signal [12], as shown in Figure 3. When the two triangular waves with the same amplitudes and slope rates are added on an integrated optic modulator (IOM), a constant frequency shift, Δf, can be introduced due to the linear phase change. The RIOG’s bias stability, $B_{s}$, was 45.144°/s, with an integration time of 10 s for 40 min. When the two triangular waves with the same amplitudes but reverse slope rates were used, the frequency difference was 2×Δf, which will reduce the interference between $I_{b}$ and $I_{s}$. The performance of RIOG was improved to 0.71708°/s, with an integration time of 10 s for 60 min. However, the static bias varies with the temperature, modulation wave, and testing environment for the lack of an effective package method.

**Figure 4:**
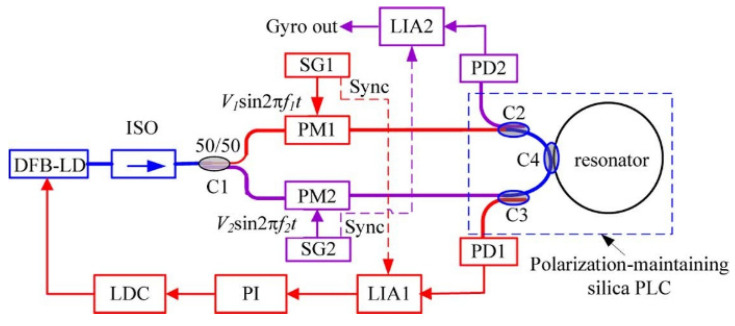
The configuration of the SPMT using the sinusoidal wave. DFB-LD: distributed feedback laser diode; ISO: isolator; LDC: laser diode controller; Sync: synchronization signal. Reprinted with permission from ref. [13]. Copyright 2011 IEEE.

Ma et al. presented a RIOG system using a sinusoidal signal based on the SPMT [13], as shown in Figure 4. The signal of sinusoidal wave modulation can be written as:

(3) $$U=V_{0}\sin(2\pi f_{m}t)$$

where $V_{0}$ and $f_{m}$ are the amplitude and frequency of the modulated signal, respectively. When a sinusoidal waveform drives the PM, the beam field at the PM output follows $E_{m}(t)=E_{0}e^{i[2\pi f_{0}t-\varphi_{0}-Msin(2\pi f_{m}t)]}$. The LiNbO_3_ modulation index, M, is expressed as $M=V_{0}\pi /V_{\pi }$, and *V_π_* is the half-wave voltage of the PD. Therefore, the laser frequency after the PM is [14]:

(4) $$f(t)=f_{0}-Mf_{m}\cos(2\pi f_{m}t)$$

The laser frequency is focused on $f_{0}$ and varies with cosine after the PM. When the laser frequency is equal to the resonant frequency of the WRR $\left(f_{o}=f_{r}\right)$, the output peak intensity is equal, and when the laser frequency departs from the resonant frequency of the WRR $\left(f_{o}\neq f_{r}\right)$, there is a height difference in the output beam intensity between the adjacent peaks. The frequency difference can be obtained by demodulating the first harmonic component of the output signal. Finally, the angular velocity of gyro rotation can be calculated. The normalized amplitude of the carrier component in the CCW direction can be defined as:

(5) $$\left|A_{0}\right|=\left|J_{0}(M)\right|$$

where M is the LiNbO_3_ modulation index, and $J_{0}(M)$ is the zeroth order Bessel function of the first kind. By suppressing the carrier component, the interference intensity between the signal light and the backscattering light can be reduced. The carrier component was decreased by nearly 100 dB by adjusting the amplitude of the sinusoidal signal, and the bias stability was increased to 0.46°/s for 50 s using a 7.9 cm-long silica WRR.

The amplitude of the modulated signal must be optimized to attain a high carrier rejection ratio. Furthermore, the PM amplitude can be optimized based on the self-heterodyne approach [71]. However, the carrier component suppression level is sensitive to the precision of the modulation index, and the half-wave voltage of the LiNbO_3_ PM is a temperature-dependent characteristic.

In SPMT, high carrier suppression levels are necessary for each PM. The modulation index is precisely adjusted to the zero-order Bessel function’s first root. The double-phase modulation technique (DPMT) is proposed to relax the precision of the modulation index. By increasing the number of phase modulators, the temperature tolerance and the modulation accuracy are improved. DPMT includes the double-phase modulation technique with the same waveform and the hybrid-phase modulation technique (HPMT) with different waveforms.

**Figure 5:**
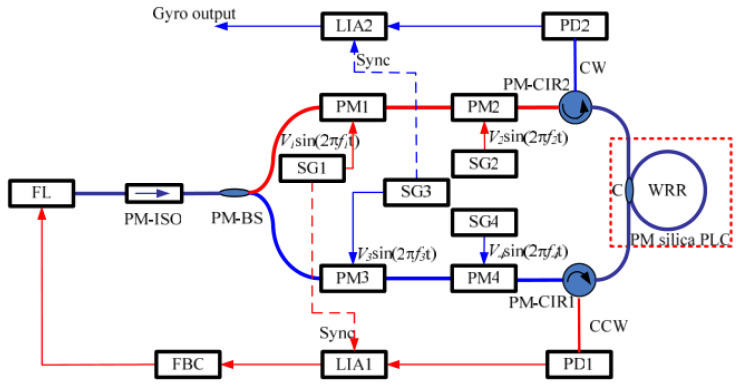
The configuration of the HPMT using the sinusoidal wave. FBC: feedback circuit; PM-ISO: polarization-maintaining isolators; PM-BS: polarization-maintaining beam splitter. Reprinted with permission from ref. [15]. Copyright 2011 The Optical Society.

Mao et al. presented DPMT with sinusoidal waves to obtain high carrier component suppression and relax the modulation index precision [21]. Figure 5 depicts the configuration of RIOG based on the DPMT. The signal modulation frequencies (*f*_1_ and *f*_3_) must be much higher than the modulation frequencies (*f*_2_ and *f*_4_). Otherwise, the extra phase modulation for carrier component suppression will weaken the gyro detection signal. The fundamental advantage of the DPMT is to reduce the remaining light-wave carrier. In the instance of the CW beam, the total carrier suppression is $J_{0}(M_{1})\times J_{0}(M_{2})$ in DPMT instead of $J_{0}(M_{1})$ in SPMT. Moreover, the CCW beam yields identical findings. The backscattering noise of the RIOG can be decreased to the order of ~10^−6^ rad/s by suppressing the carrier component voltage at about 10^−2^. Then, the backscattering noise is less than the ultimate sensitivity of the RIOG, $\Omega_{\min}(\sim 7.9\times 10^{-5}\mathrm{rad}/s)$. Therefore, the precision of the modulation index is relaxed. The RIOG based on DPMT can achieve bias stability of 3.14 × 10^−3^ rad/s.

**Figure 6:**
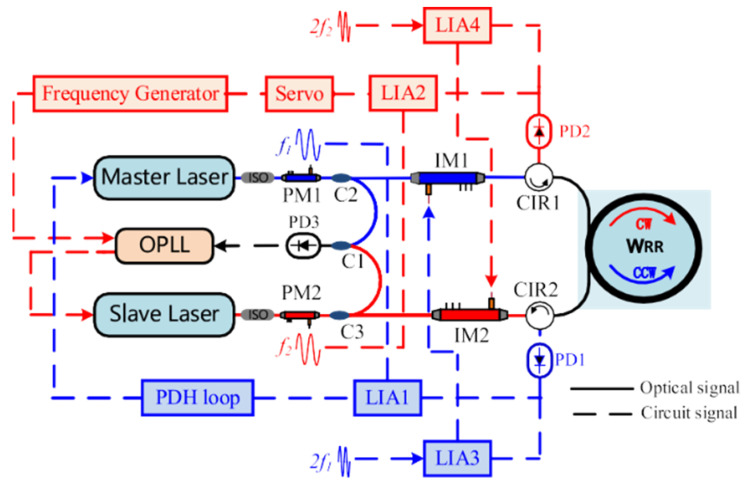
The configuration of the RIOG based on double light sources. PDH: Pound–Drever–Hall; ISO: isolator; IM: intensity modulator. Reprinted with permission from ref. [22]. Copyright 2021 Elsevier.

Niu et al. proposed a suppression approach based on double light sources [22], in which light for CW and CCW is provided by two tunable semiconductor lasers, respectively, as shown in Figure 6. Optical Phase-Locked Loops (OPLL) lock the operating frequencies of the two separate lasers to the WRR’s CW and CCW resonant frequencies. High-frequency noise induced by interference (see Equations (1) and (2)) between the backscattering light and the signal light can be eliminated in this manner. Adjusting the OPLL bandwidth lowered the frequency noise for double lasers as well. Then, intensity modulators with second harmonic feedback were used to stabilize the optical power in the cavity. Compared with the SPMT in a single-laser system, the impact of interference light was decreased by order of magnitude, and the bias stability was 0.00448°/s of a 5 s integration time.

### 3.2. Back-Reflection Noise

#### 3.2.1. The Back-Reflection Noise

Unlike the backscattering noise, the back-reflection appears at the medium interface outside the WRR, including the end faces and defects of waveguides. For RIOG, where all components are integrated onto a single substrate, the back-reflection should be a minimum. However, for the current hybrid integration, the most explored low-loss material for the WRR is the silica PLC, and phase modulators are to be formed in high-performance LiNbO_3_ materials. Fibers are usually used to butt joints between the PM and WRR. Strong back-reflection can occur at the interface between an optical fiber and a straight waveguide. The out-cavity back-reflection causes severe degradation on the RIOG [23,72]. Back-reflection noise is commonly decreased by beveling the end faces of components [73] or placing isolators ahead, and the back-reflection coefficient can reduce to about −60 dB [74]. According to the two-beam interference theory [75], the back-reflection will produce a ripple on the photocurrent signal with a maximum peak-to-peak value of −24 dB, which should not be overlooked in RIOG. Since further reducing the back-reflection coefficient is too challenging, new ways to suppress back-reflection noise should be investigated.

#### 3.2.2. Suppression Method of Back-Reflection Noise in RIOG

Angle polishing has dramatically reduced the amplitude of back-reflection noise, but it is still a significant factor in RIOG.

Commonly, phase modulation technology adjusts the bias operation point to improve detection sensitivity while suppressing the back-reflection- and backscattering-induced errors [10,13,35]. If phase modulation technology is used, the back-reflection noise will cause more significant deformation of the resonator’s initial output signal [26]. Since the signal bandwidth is generally less than one kilohertz, and the resonator’s initial output signal frequency is hundreds of kilohertz, the extra deformation does not affect the gyro’s long-term bias stability [25]. By oversampling and applying a mean filter, the high-frequency noise induced by additional deformations can be significantly reduced and has little impact on the gyro bias drift.

**Figure 7:**
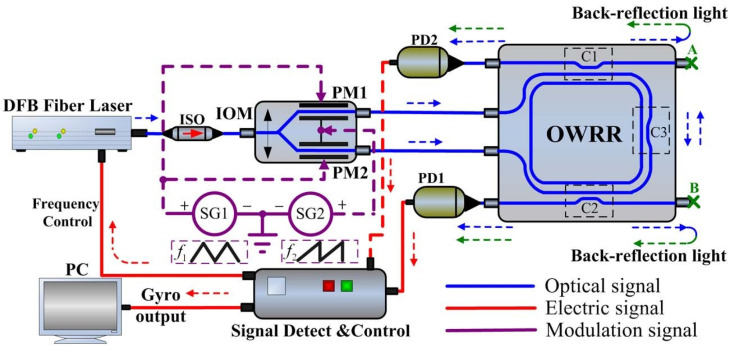
Schematic illustration of the RIOG with two facet-reflection dots. OWRR: optical waveguide ring resonator. Reprinted with permission from ref. [24]. Copyright 2013 The Optical Society.

RIOGs have a wide dynamic range in rotation sensing, reaching 1.4 × 10^4^ rad/s [76], larger than the equivalent frequency shift induced by the sawtooth wave. The scale factor and nonlinearity would not be affected by HPMT. Under hybrid phase modulation, the back-reflection noise spectra will separate from one another. Back-reflection noise can be successfully minimized when used in conjunction with the pectinate-filter properties of digital correlation detection. HPMT is a viable method for suppressing the back-reflection noise by simultaneously adding serrodyne and triangular waves onto the PM [24], as shown in Figure 7. Compared with triangular phase modulation technology [12], the bias stability has improved dramatically, from 2.34 to 0.22°/s with a 10 s integration time.

**Figure 8:**
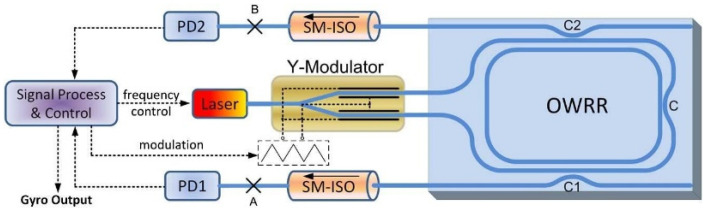
Sketch map of a RIOG system. SM-ISO: single-mode isolator; solid line, optical circuit; dash line, electric circuit. Reprinted with permission from ref. [26]. Copyright 2013 The Optical Society.

The method proposed by Wang et al. eliminates back-reflection-induced sampling errors in digital correlation detection by employing integer period sampling (IPS) [26], as shown in Figure 8. As a result of back-reflection and signal light interfering, a cosine is formed—the unwanted cosine wave results in severe sampling errors. When the IPS approach was used to decrease the sampling error for suppressing back-reflection in RIOG, both the short- and long-term bias stabilities improved once the IPS condition was satisfied. Long-term bias stability of 0.41°/s was achieved with a 10 s integration time, and short-term bias stability of 0.067°/s was achieved with a 10 s integration time.

**Figure 9:**
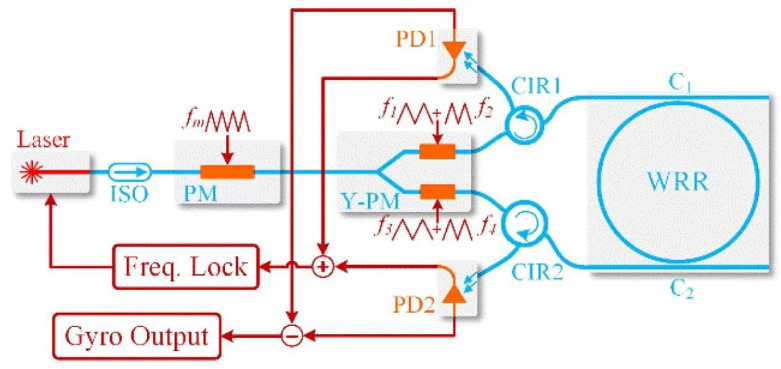
Schematic diagram of the RIOG with EDDT. Y-PM: Y-branch phase modulator; CIR: circulator. Reprinted with permission from ref. [27]. Copyright 2018 The Optical Society.

Wang et al. proposed an enhanced differential detection technique (EDDT) [27] for suppressing common-mode signals and improving the RIOG’s detection accuracy, as shown in Figure 9. It should be emphasized that differential-mode signal distortion is induced by backscattering and back-reflection errors. A unique construction based on a transmissive resonator has increased the reciprocity of the RIOG, which benefits from decreasing the expected error. The differential mode output of the RIOG is proportional to the angular rotation of the gyro. The common-mode rejection ratio can be improved by suppressing the common-mode signal of RIOG. The EDDT technique successfully achieves long-term bias stability of 0.0029°/s over two hours.

**Figure 10:**
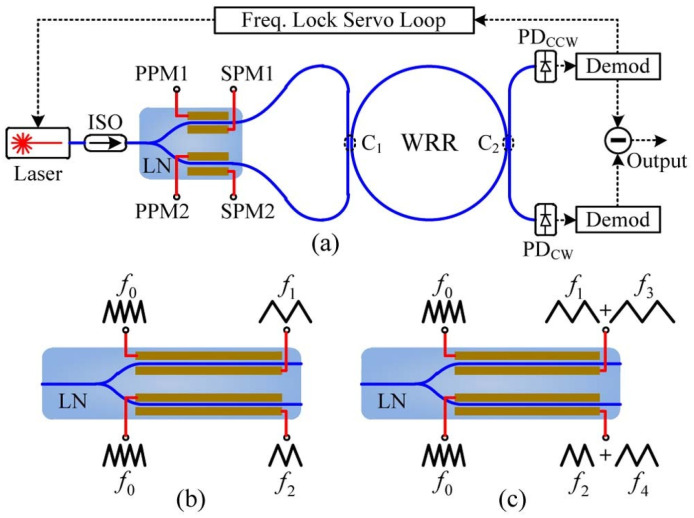
(**a**) Schematic diagram of a RIOG using PDT. (**b**,**c**) Two types of PDT implementations. LN: lithium niobate modulator; SPM: secondary phase modulation; PPM: primary phase modulation; Demod: demodulation. Reprinted with permission from ref. [28]. Copyright 2016 The Optical Society.

The phase difference traversal (PDT) method is proposed by Feng et al. to suppress the back-reflection noise in RIOG [28], as shown in Figure 10a. The back-reflection-induced error can be effectively suppressed by making the phase difference between the CW and CCW incident light traverse the interval [0, 2π] repeatedly and rapidly enough [28]. An in-phase modulation approach can significantly reduce back-reflection ripples and minimize the gyro’s angle random walk [25]. The primary and secondary phase modulation signals are grounded and connected to the Y-branch phase modulator for simultaneous use, as shown in Figure 10a. The phase difference traversal method adjusts the frequency spectrum of the back-reflection-induced error signal from low to high, thereby reducing the spectral overlap between the back-reflection-induced error signal and the rotation speed signal. Since different secondary phase modulation signals change rapidly, this results in a further reduction of the spectrum overlap and an increase of the PDT method’s accuracy tolerance; therefore, the multi-wave hybrid phase modulation method shown in Figure 10c is better in back-reflection suppression than the scheme shown in Figure 10b. A short-term bias stability of 0.0055°/s was reached in 5 min, while a long-term bias stability of 0.013°/s was achieved in 1 hour. The Allan deviation [77] analysis of a typical one-hour test shows that bias stability had reached 0.006~0.007°/s. The peak-to-peak value of zero bias is less than 0.1°/s, which is probably due to residual back-reflection, Kerr effect, polarization drift, and light intensity fluctuation.

### 3.3. Optical Kerr Effect

#### 3.3.1. Nonlinear Kerr Noise

The Kerr effect is a third-order nonlinear phenomenon, and the light intensity affects the refractive index [78]. Therefore, a phase delay occurs when the light passes through a medium with the Kerr effect. The Kerr effect-induced bias error is proportional to the light intensity difference between the CW and CCW lightwaves in the resonator [29,30,31,79], and the light intensity fluctuations would influence the long-term bias stability of the RIOG. The presence of two counterpropagating waves causes the formation of a nonlinear refractive index grating [80]. If the light intensities of the CW and CCW lightwaves differ, a small nonreciprocal phase error will result in a nonzero bias at the gyro output [29,30,31]. Therefore, fluctuations in light intensity affect the gyro bias stability.

For a RIOG with high sensitivity, the Kerr effect causes the drift to be far more than the theoretical limit of rotation sensitivity provided by the detector shot-noise. A slight imbalance of 0.01% between the two lightwaves can cause a bias of two orders of magnitude larger than the shot-noise limited theoretical sensitivity [31]. The optical Kerr noise is slight compared with other noises [33], such as backscattering, back-reflection, and polarization fluctuation. However, countermeasures need to be taken for high-performance RIOG.

#### 3.3.2. Suppression Method of Kerr-Induced Noise in RIOG

**Figure 11:**
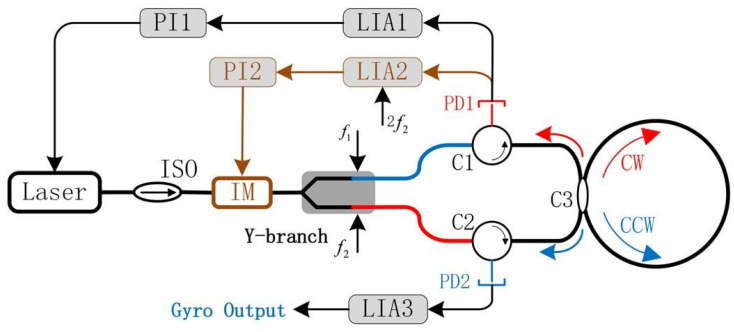
The basic configuration of the RIOG system with light intensity feedback. Reprinted with permission from ref. [34]. Copyright 2020 SPIE.

The intensities of the CW and CCW lightwaves circulating in the resonator should always be equal to eliminate drifts caused by the optical Kerr effect. In a meter-scale RFOG, a square wave with a duty cycle of 50% can be used as the signal of intensity modulation to suppress the optical Kerr effect noise. The square-wave intensity modulation is available for the meter-scale RFOG and not for the centimeter-scale RIOG due to the square-wave frequency being an integer multiple of the resonator’s free spectral range [29]. A light intensity feedback loop technique was proposed by Yin et al. to stabilize the light intensity input into the gyroscope system [34], as shown in Figure 11. The light intensity feedback loop consists of LIA2, PI2, and an intensity modulator (IM). The maximum value of the second harmonic demodulation curve is used to track the light intensity, while PI2 controls the IM to stabilize the light entering the Y-branch, forming the light intensity feedback loop. The nonlinear error of the system scale factor was reduced from 13.74% to 2.79%, and the gyroscope’s dynamic performance was improved. At the same time, the long-term bias stability of 16.94°/h in one hour was achieved.

**Figure 12:**
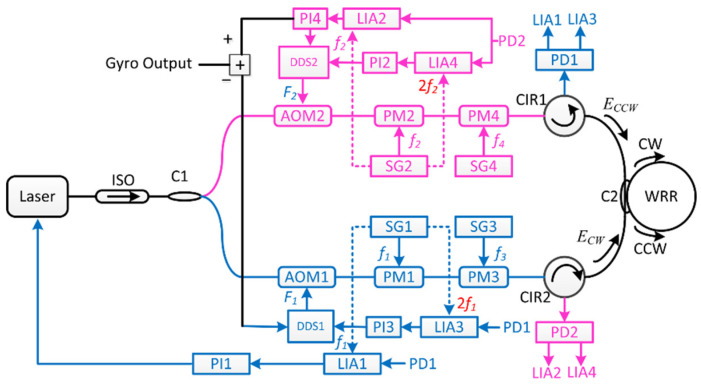
Schematic diagram of the RIOG with the light intensity feedback loop. Reprinted with permission from ref. [35]. Copyright 2014 The Optical Society.

For the sinusoidal phase modulation, Ma et al. demonstrated that the second-harmonic demodulated signal is proportional to light intensity in the WRR [35]. The demodulated second-harmonic signal can be employed as a feedback error signal to decrease the input-intensity mismatch and intensity fluctuations between the CW and CCW lightwaves. Two acousto-optic modulators (AOMs) as two frequency shifters are shown in Figure 12. The direct digital synthesis (DDS) module generates tunable sinusoidal signals for adjusting the frequency and intensity after the light passes through the acousto-optic modulator. The feedback signals from LIA3 and LIA4 are utilized to modulate the amplitude of the driving signals from the two direct digital syntheses. The light intensity fluctuation can be reduced by adjusting the light intensity input to WRR in real-time through the light intensity servo circuit. The light intensity feedback loop reduces light intensity fluctuations to 2.7 × 10^−5^, down from 5.86%.

**Figure 13:**
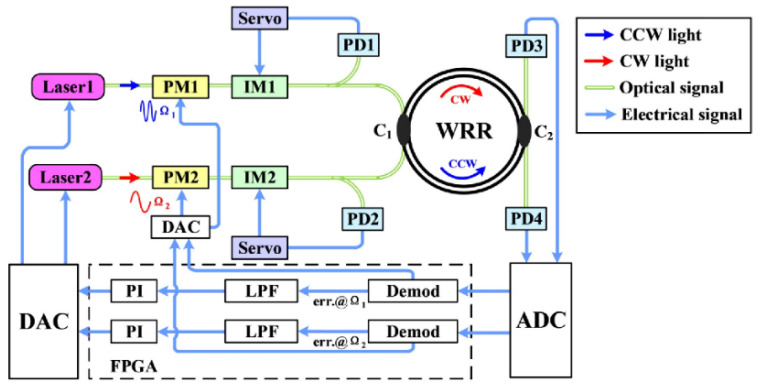
Schematic diagram of the RIOG with two independent lasers. DAC: digital-to-analog converter; FPGA: field programmable gate array; LPF: low-pass filter. Reprinted with permission from ref. [36]. Copyright 2021 The Optical Society.

Niu et al. proposed a method to suppress backscattering noise and Kerr noise in a RIOG [36], as shown in Figure 13. It employs two independent lasers to lock the CW and CCW optical signals at different frequencies, effectively suppressing backscattering noise and resulting in a differential output. Simultaneously, a light intensity feedback loop based on a light intensity modulator is added to the loop to ensure consistent optical power to reduce Kerr noise. The light intensity fluctuation decreased by two orders of magnitude, and the gyro’s bias stability was increased to 9.06°/h. 

### 3.4. Polarization Noise

#### 3.4.1. Nonreciprocal Polarization Fluctuation Noise

A birefringence polarization-maintaining resonator has two polarization modes, which duplicate their polarization states after one round-trip through the resonator [38]. The unwanted eigenstates of polarization (ESOP) appear as the second peak or dip in the resonant curve [38]. The temperature affects the birefringence of the polarization-maintaining WRR. Therefore, the polarization fluctuation error is affected by environmental temperature changes, which affect the long-term stability of the gyros [41].

The polarization fluctuation noise is a nonreciprocal error that cannot be reduced by the digital PI controllers [81,82]. It is caused by the intensity-type noise [30] and interference-type noise [83], which causes the resonance curve to become asymmetric and the frequency difference measurement error to rise. Fixing an unwanted resonance angle at the center of another resonance interval for rotation induction can suppress the intensity noise [84]. However, when there is a polarization-dependent loss in the resonator, the suppression method of the intensity noise cannot effectively reduce the interference noise [40]. Twin 90° polarization-axis-rotated splices in a resonator are proposed for reducing polarization fluctuation-induced noise in RFOG [40]. The TM mode’s resonance curve can be inhibited by applying a polarization controller [45] before the line enters the WRR. However, this technology is challenging to implement in RIOG.

#### 3.4.2. Suppression Method of Polarization Fluctuation-Induced Noise in RIOG

**Figure 14:**
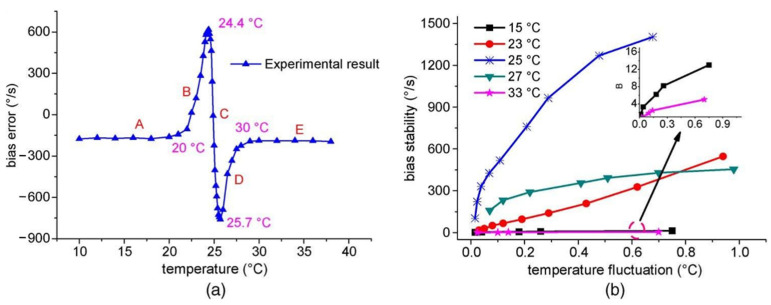
Experimental findings of (**a**) bias error of the RIOG in different temperatures and (**b**) bias stability of the RIOG in different temperature fluctuations. Reprinted with permission from ref. [43]. Copyright 2012 IEEE.

Yang et al. tested the polarization fluctuation-induced drift for different temperatures [41]. The experimental results show that a suitable temperature can improve the gyro’s long-term stability. For the WRR, a suitable temperature can adequately separate two ESOPs. For example, when the temperature is near 24 °C, a 0.03 °C fluctuation results in a 65°/s output error. The influence of temperature changes on the gyro’s bias stability is minimized when the temperature of the WRR is lower than 16 °C or greater than 33 °C, as illustrated in Figure 14a. As demonstrated in Figure 14b, setting a suitable temperature can significantly decrease polarization fluctuation noise and improve the gyro’s output stability. In practical application, the working environment temperature of the gyroscope cannot be controlled.

**Figure 15:**
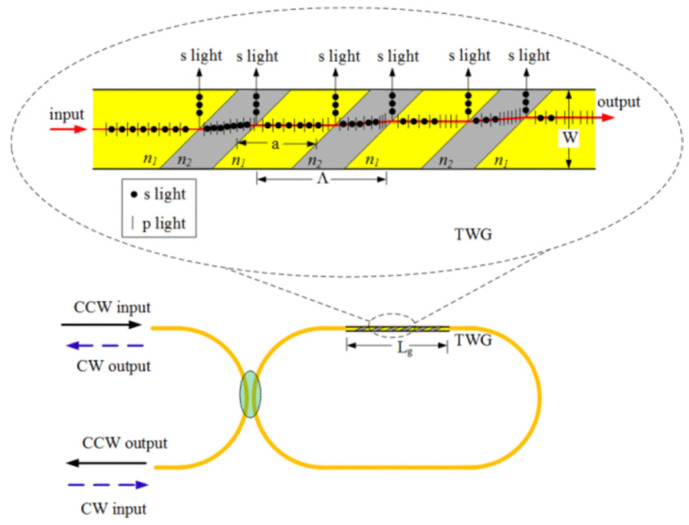
The WRR with a 45° tilted waveguide grating. Reprinted with permission from ref. [46]. Copyright 2015 IEEE.

Improving the WRR’s polarization extinction ratio (PER) is an appealing alternative method. The in-cavity and out-cavity methods are two methods for improving the WRR’s PER [85]. As shown in Figure 15, the tilted waveguide gratings (TWGs) with Brewster’s angle were presented for the silica WRR [46], which is an in-cavity method with the most perceptible improvement [50]. The in-cavity PER can reach 40 dB by adding a 4 cm-long 45° tilted grating to the original WRR and using the best structure parameters. However, the fabricated process is not mature at present [48].

**Figure 16:**
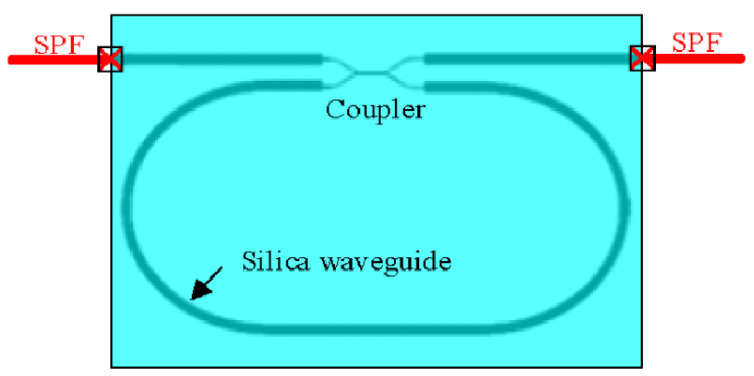
Schematic diagram of the WRR with a single-polarization fiber. Reprinted with permission from ref. [49]. Copyright 2017 The Optical Society.

The out-cavity method is more accessible to implement than the in-cavity method described above. A secondary eigenstate of polarization (S-ESOP) in the WRR and a second resonance dip cause the polarization problem. As shown in Figure 16, WRR coupling with a single-polarization fiber (SPF) can lower the amplitude of the S-ESOP. Compared with the polarization-maintaining fiber pigtail, the PER of the single polarization pigtail is increased from 10.7 to 18.90 dB [49]. The WRR attained finesse and resonant depths of 196.7% and 98%, respectively. Over a one-hour timeframe, bias stability of 0.004°/s was obtained using this high-finesse and high-PER WRR [49].

**Figure 17:**
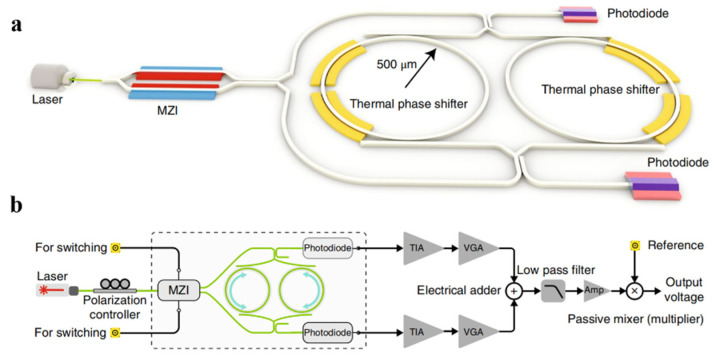
(**a**) Schematic of the implemented nanophotonic optical gyroscope. (**b**) System architecture with electronic circuitry. Signals from two paths are added together and multiplied by the reference frequency through a passive mixer to extract the amplitude information that encodes the rotation rate. TIA: transimpedance amplifier; VGA: variable gain amplifier; Amp: amplifier. Reprinted with permission from ref. [86]. Copyright 2018 Nature Photonics.

These temperature fluctuations in silicon nanophotonics have a bandwidth of several kilohertz, are much less pronounced above a megahertz, and thus can be treated as constant over timescales of microseconds. Khia et al. developed an approach called reciprocal sensitivity enhancement [86], as shown in Figure 17. In a passive network made of isotropic elements (lossy or lossless), switching the input and output ports does not change the observed response from input to output. This approach reduces fluctuation noise because of the temporal separation between the CW and CCW propagating beams in each path and the cancellation of thermal fluctuations using high-frequency optical switching. Critically, this method is more tolerant to the propagation loss of the medium. Using a Mach–Zehnder interferometer (MZI), the ring resonators can be fed from two different directions, and the output toggles between two photodiodes. By adding the two outputs together, information ‘moves’ to the switching frequency and becomes unaffected by thermal drift. The all-integrated optical gyroscope occupies only 2 mm^2^ and detects the smallest recorded phase shift (3 nrad).

### 3.5. Laser Frequency Noise

Aside from the noise concerns mentioned above, another critical precision limiting element could be laser frequency noise [52,53]. When the laser frequency is changed for frequency locking, the laser’s stability will be decreased. The laser bandwidth will be stretched out much further, resulting in poor accuracy of the RIOG. The intensity noise is manifested as laser intensity variation, which causes the output inaccuracy in RIOG. The laser intensity-induced error is proportional to the nonzero bias between the CW and the CCW resonant frequencies [87]. Laser frequency noise above the rotation-rate detection band could be filtered away. However, some actual experiment results revealed that frequency noise at the high-frequency range on the laser performance seems far beyond that at the low-frequency range [83].

Duan et al. proposed a mothed of frequency locking inside the WRR using a piezoelectric fiber PM [52]. The influence generated by laser linewidth spreading is minimized by locking frequency in the resonator, which helps increase the gyro’s bias stability. A transmission-type RFOG was built using a PZT cylinder for frequency locking. Angle random walk was decreased from 0.0069 to 0.0050°/√h. Sanders et al. recognized high-frequency laser noise as a problem and presented a solution using an optical filter and laser stabilization [88]. The optical filter and laser stabilization could attenuate the laser frequency noise by 10 dB at the second harmonics of the bias modulation frequency. Unfortunately, they have not yet been applied to adjust the resonant frequency of WRR in RIOG.

## 4. New Noise Suppression Technology

In recent years, leading research has been invested in enhancing the Q-factor of the WRR. On the other hand, multi-beam interference light is a weak signal in angular velocity information due to noise, optical nonlinearity, and parameter uncertainty. Since the Sagnac frequency difference is proportional to the ratio of the area and perimeter of the ring resonator, a slight rotation angular velocity only corresponds to a small resonant frequency difference. Therefore, signal detection technology is critical for improving performance in practical engineering applications.

**Figure 18:**
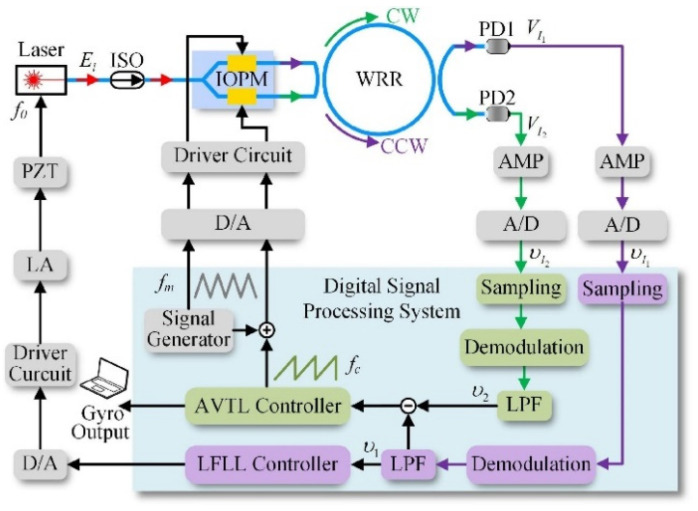
The signal detection scheme of the RIOG system. IOPM: integrated optical phase modulator; LA: linear amplifier; PZT: piezoelectric transducer; LFLL: laser frequency lock loop; AVTL: angular velocity tracking loop. $f_{m}$ is the frequency of triangular modulation, and $f_{c}$ is the frequency of the feedback sawtooth wave. Reprinted with permission from ref. [89]. Copyright 2021 IEEE.

Li et al. presented a closed-loop signal-detecting approach to increase the RIOG’s dynamic performance [89], as shown in Figure 18. A control algorithm is presented to reduce the impact of optical nonlinearity and parameter uncertainty on RIOG’s dynamic tracking performance. The signal-to-noise ratio (SNR) of the closed-loop error signal of angular velocity tracking can be improved by optimizing the signal-processing loop gains. The gyroscope based on the control algorithm has good robustness to suppress optical power fluctuation. The experimental results show that the rise time of the RIOG is less than 36 μs.

**Figure 19:**
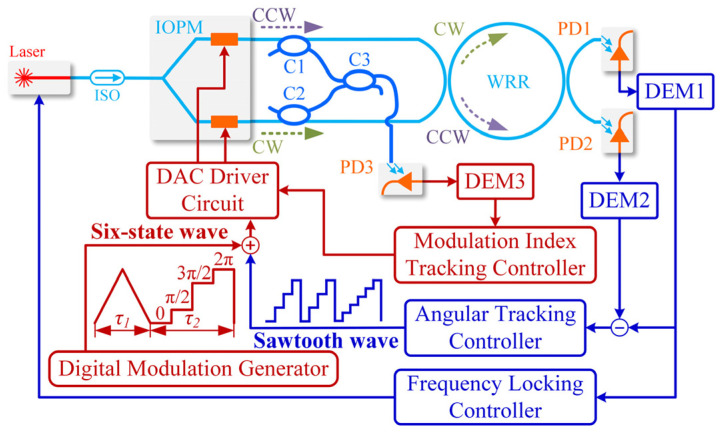
The schematic diagram of RIOG with the modulation index stabilization technique. DEM: demodulator; C1: 99:1 optical coupler; C2: 99:1 optical coupler; C3: 50:50 optical coupler. Reprinted with permission from ref. [90]. Copyright 2019 The Optical Society.

Wang et al. proposed a new modulation index stabilization technique (MIST) to track the integrated optic phase modulator (IOPM). It can enhance the performance of temperature stabilization in RIOG [90], as shown in Figure 19. The gyro scale factor stability from the IOPM’s modulation index fluctuation was attained at 189.26 ppm within −40 to +60 °C by real-time demodulation and modification of the modulation index of IOPM.

**Figure 20:**
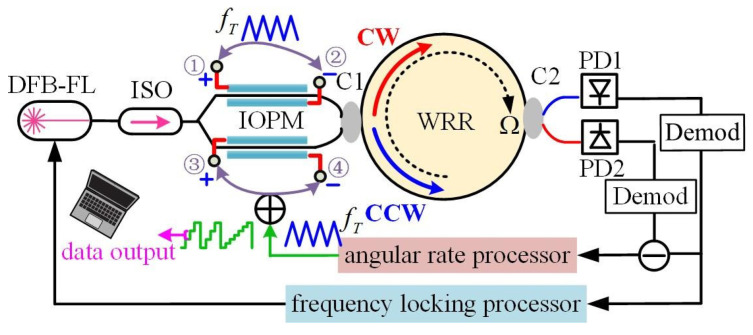
The schematic diagram of RIOG with the double closed-loop control system. 1 and 2 are the upper arm of the IOPM, 3 and 4 are the bottom arm of the IOPM. Reprinted with permission from ref. [91]. Copyright 2018 The Optical Society.

The locking-frequency precision of the laser frequency-locking loop significantly impacts the performance of the main angular velocity tracking loop in a practical RIOG system. Meanwhile, various negative variables such as the optical effect, optical parameter fluctuation, and the external noise present in the actual environment degrade the detection accuracy of RIOG. Li et al. developed a double closed-loop control system of the mean-square exponential stable to improve the detection accuracy and dynamic response characteristics of RIOG [91], as shown in Figure 20. The triangular wave for phase modulation is applied on the angular velocity tracking loop (IOPM). The sawtooth feedback wave is applied on the bottom arm of the IOPM. Meanwhile, the phase modulation triangular wave and digital feedback sawtooth wave are differentially applied on the arms of the IOPM. The IOPM achieves phase modulation and enables the angular velocity tracking loop (AVTL). The RIOG has a response time of less than 76 μs. Long-term bias stability of 7.04°/h in one hour was achieved in RIOG.

**Figure 21:**
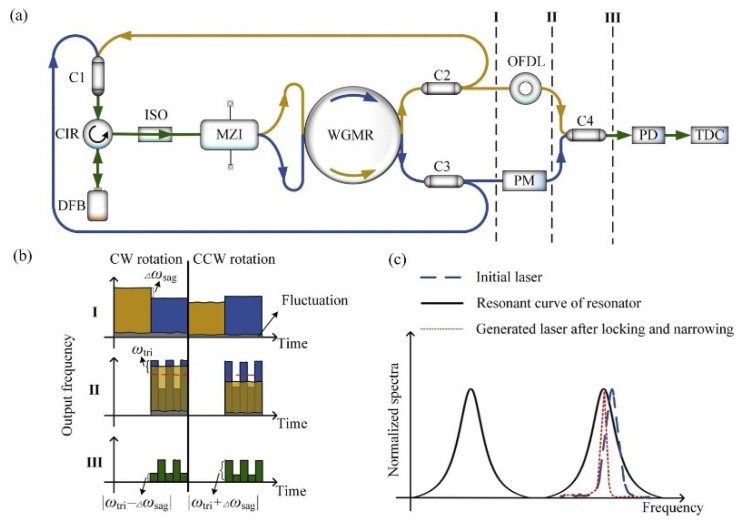
Configuration of RIOG. (**a**) The configuration of RIOG with the self-injection locking technique. (**b**) The output from the laser is switched between the yellow path and blue path at parts I and II, and a beat frequency signal is formed at part III. (**c**) Scheme of frequency-locking and linewidth narrowing. Reprinted with permission from ref. [92].Copyright 2020 The Optical Society.

Yang et al. have presented a novel RIOG based on a self-injection locking technique [92]. The drift of laser frequency and disturbance of resonant frequency induced by temperature fluctuations can be eliminated by detecting the beat frequency. The absolute frequency difference is the beat frequency that combines the two light fields (from the yellow and blue paths shown in Figure 21a). The core frequency is locked to the resonator mode via self-injected locking technology, as shown in Figure 21c. The rotating direction of RIOG can be distinguished easily by beat frequency $\left|\Delta \omega_{sag}\pm \omega_{tri}\right|$, where $\Delta \omega_{sag}$ is the frequency difference caused by the Sagnac effect, and $\omega_{tri}$ is the triangular wave-modulated frequency by PM, as shown in Figure 21b. The gyro’s sensitivity is significantly improved by increasing reciprocity and monitoring beat frequency, and theoretical sensitivity is demonstrated to reach 10^−4^°/h under a 6 kHz modulation frequency.

Most digital signal generation and processing in the existing RIOG are based on field programmable gate arrays (FPGA) [93,94]. Their strong logic, fast speed, and high efficiency have more apparent advantages in signal generation and processing. In the traditional software operation, the data are ready before processing. FPGA can receive simultaneous multichannel data from the sensor and process it in real time.

A high-precision digital signal processor using an FPGA circuit is essential for the accuracy of RIOG. There are many new digital signal processing applications in the noise suppression system. A digital detection technique based on the coordinate rotation digital computer (CORDIC) [65] algorithm was presented by Yang et al. to generate sinusoidal waves. It means the modulation signal generation, synchronous demodulation, and signal processing are realized in a single FPGA. The synchronous digital quadrature demodulation technique [95] was introduced by Wu et al. Compared with the common sinusoidal demodulation technique, the performance of the quadrature demodulation technique has advantages in accurate phase alignment and system phase noise suppression. Therefore, using FPGA for signal processing is convenient for applying new noise suppression algorithms.

## 5. Summary of Noise Suppression

Several main noise suppression methods are compared in Table 1. In RIOG, optical backscattering noise is generally considered the most significant noise. Phase modulation technology is widely used as the basic technology of suppressing backscattering noise in RIOG. As a first-generation suppression technology, SPMT is simple and suffers lower optic loss. However, this technology has an inevitable drawback. The carrier suppression level is susceptible to the accuracy of modulation amplitude. The carrier suppression will be decreased when the temperature drifts. Several phase modulation techniques have been proposed to improve detection sensitivity, including sinusoidal modulation, triangular modulation, serrodyne modulation, and hybrid modulations. Hybrid modulation can be used to achieve high carrier suppression, and backscattering error can be reduced to the level below the shot-noise limited sensitivity of the RIOG. The requirements for modulation amplitude accuracy and temperature stability are also remarkably relaxed. However, the technique makes the resonator’s response more complicated. More waveforms will be applied to phase modulation in the future, and better results may appear.

By suppressing common-mode signals of backscattering and back-reflection noise, excellent long-term bias stability of 0.0029°/s was achieved [27]. As is shown in Table 1, the best short-term bias stability of 0.0055°/s is achieved by the phase difference traversal method. The light intensity feedback loop technique has been applied to eliminate the drifts due to the optical Kerr effect. Dual light sources and a light intensity feedback loop based on a light intensity modulator are added to ensure the same optical power for CW and CCW lightwaves to reduce Kerr noise. In the method of suppressing Kerr noise, the bias stability of 9.06°/h is the best result of long-term bias stability. The light intensity feedback loop technology can also eliminate the Kerr noise due to the intensity variations of the laser. Setting an appropriate temperature and improving the PER is the primary suppression method of polarization noise. There are two methods to improve the PER of the WRR, i.e., the in-cavity method and the out-cavity method. The WRR coupling with the SPF can achieve bias stability of 0.004°/s. With the mature methods for these noise factors, laser frequency noise has become a new accuracy limiting factor. When the laser frequency is changed to be frequency-locked, the laser stability will be lessened, and the laser bandwidth will be further expanded, resulting in the low accuracy of the RIOG. Unfortunately, there is no practical way to suppress laser frequency noise in RIOG. In recent years, more and more gyroscopes based on new structures have been proposed, but the optical noises are still essential obstacles limiting the performance of gyroscopes. To our knowledge, a double closed-loop control system with the bias stability of 7.04°/h is the best result of long-term bias stability [91]. Silicon nanophotonics technology is an ideal platform to realize an integrated optical gyroscope because of its reliability and compatibility with current mature production technology. In addition, it can integrate nanophotonics and electronic components into a single substrate. Optical gyroscopes are very suitable for miniaturization on the nanophotonic platform. However, the SNR of the optical gyroscope is usually limited by optical noise. Due to the relatively weak signal intensity, the integrated optical gyroscope has not been realized. In the future, new noise suppression technologies are needed to comprehensively reduce all kinds of noise and improve the short- and long-term bias stability.

## 6. Conclusions

Much progress on RIOG has been made in recent years. A high-performance RIOG requires a signal detection technique with a high signal-to-noise ratio and accuracy. Phase modulation technology is widely used as the mature technology of suppressing backscattering and back-reflection noise in RIOG. Dual light sources and light intensity feedback loop technology can make the intensities of the CW and CCW lightwaves equal to reduce Kerr noise. Improving the PER of the WRR with an in-cavity method is the most noticeable improvement. However, there is no practical way to suppress laser frequency noise in RIOG. This paper summarizes and compares the existing systems of RIOG. The dual-laser and double closed-loop signal detection technology are likely to be the methods bysuppressing various noises in RIOG. Implementing digital signal processing in FPGA will be the future trend, which is convenient for applying signal processing algorithms in RIOG. It is still a great challenge to integrate all components on a single chip by monolithic integration. Therefore, hybrid integration is the only option at present. Hybrid integration allows all elements of the system to be optimized through the best technical solution. Overall, the existing RIOGs are still far from the on-chip integrated gyro. We need to make more efforts to realize RIOGs with small size and high accuracy. 

## Figures and Tables

**Table 1:** Main noise suppression technologies.

Noise	Technology	Short-Term Bias Stability	Long-Term Bias Stability
backscattering noise	SPMT with triangular wave [12]	-	0.71708°/s
	SPMT with sinusoidal wave [13]	0.46°/s	-
	DPMT with sinusoidal wave [21]	3.14 × 10^−3^ rad/s	-
	dual light sources [22]	-	0.00448°/s
back-reflection noise	HPMT with triangular wave and sawtooth wave [24]	-	0.22°/s
	integer period sampling [26]	0.067°/s	0.41°/s
	enhanced differential detection technique [27]	-	0.0029°/s
	phase difference traversal [28]	0.0055°/s	0.013°/s
Kerr noise	light intensity feedback loop [34]	-	16.94°/h
	HPMT and the light intensity feedback loop [35]	-	-
	dual light sources and light intensity feedback loop [36]	-	9.06°/h
polarization noise	tilted waveguide gratings [46]	-	-
	single-polarization fiber [49]	-	0.004°/s
	reciprocal sensitivity enhancement [86]	-	-
laser frequency noise	-	-	-
new noise suppression technology	closed-loop signal detection [89]	-	-
	modulation index stabilization technique [90]	-	-
	double closed-loop control system [91]	-	7.04°/h
	self-injection locking technique [92]	-	-

## Data Availability

Not applicable.
